# Mechanistic Insights Delineating the Role of Cholesterol in Epithelial Mesenchymal Transition and Drug Resistance in Cancer

**DOI:** 10.3389/fcell.2021.728325

**Published:** 2021-11-19

**Authors:** Naaziyah Abdulla, C. Theresa Vincent, Mandeep Kaur

**Affiliations:** ^1^ School of Molecular and Cell Biology, University of the Witwatersrand, Johannesburg, South Africa; ^2^ Department of Immunology, Genetics and Pathology, Uppsala, Sweden; ^3^ Department of Microbiology, New York University School of Medicine, New York, NY, United States

**Keywords:** cholesterol, drug resistance, hypoxia, EMT, cancer

## Abstract

Despite the significant advancements made in targeted anti-cancer therapy, drug resistance constitutes a multifaceted phenomenon leading to therapy failure and ultimately mortality. Emerging experimental evidence highlight a role of cholesterol metabolism in facilitating drug resistance in cancer. This review aims to describe the role of cholesterol in facilitating multi-drug resistance in cancer. We focus on specific signaling pathways that contribute to drug resistance and the link between these pathways and cholesterol. Additionally, we briefly discuss the molecular mechanisms related to the epithelial-mesenchymal transition (EMT), and the documented link between EMT, metastasis and drug resistance. We illustrate this by specifically focusing on hypoxia and the role it plays in influencing cellular cholesterol content following EMT induction. Finally, we provide a proposed model delineating the crucial role of cholesterol in EMT and discuss whether targeting cholesterol could serve as a novel means of combatting drug resistance in cancer progression and metastasis.

## Introduction

Cancer has been predicted to be the leading cause of death in every country in the world in the 21st century (Bray et al., 2018). In 2020, an estimated 10 million deaths were accredited to cancer with approximately 70% of these deaths documented in low- and middle-income countries (Sung et al., 2021). The International Agency for Research on Cancer predicted a worldwide increase in cancer cases from 18,078 957 (2018) to 28,400 00 in the year 2040, while a 78% increase in cancer cases in South Africa has been predicted by the year 2030 (Bray et al., 2018; Sung et al., 2021). Even though scientific advances in cancer research continue to provide renewed hope to cancer patients with major improvements documented in surgery, multidrug resistance (MDR) constitutes a major hurdle leading to therapy failure, and ultimately contributing to over 90% of cancer patient deaths (Bukowski et al., 2020). Intrinsic drug resistance occurs when the tumor cell mass present with resistant mechanisms prior to therapy administration, while acquired resistance describes a condition where cells that were initially sensitive to therapy develop mechanisms that confer them with survival traits during therapy (Haider et al., 2020). Multiple mechanisms implicated in conferring drug resistance to cancer cells have been documented in literature which include but are not limited to: enhanced drug efflux capability, mutations to the drug targets, increased metabolism of xenobiotics leading to drug detoxification and concomitant inactivation, hyperactivation of survival pathways coupled with inactivation of apoptotic pathways as well as the induction of the epithelial-mesenchymal transition (EMT) (Housman et al., 2014; Du and Shim, 2016; Ke and Shen, 2017; Bukowski et al., 2020; Haider et al., 2020). Importantly, the acquisition of resistance is dictated by inherent genetic instability, mutagenicity of tumor cells as well as the influence of cells in the tumor microenvironment (Haider et al., 2020). Increasingly now, emerging experimental evidence highlight the crucial role of cholesterol metabolism in facilitating drug resistance in cancer. This review aims to address the complexities underlying the role of cholesterol in facilitating MDR in cancer cells. Additionally, we document the link between EMT, metastasis and drug resistance, and illustrate this by specifically focusing on hypoxia as an extrinsic factor that influences cholesterol to facilitate EMT-induced drug resistance. This is followed with an accompanying discussion and a proposed hypothesis concerning whether targeting cellular cholesterol may serve as a novel strategy for combating cancer progression.

### Role of Cholesterol in a Cell and Mechanisms of Cholesterol Homeostasis

Cholesterol is a crucial lipid known to maintain cellular homeostasis by regulating the survival and growth of cells (Kuzu et al., 2016). It also serves as the key sterol component in the plasma membrane constituting approximately 30% of the lipid bilayer (Zhang J. et al., 2019). Structural integrity and fluidity of the cell membrane is thus consequently dictated by membrane cholesterol content. As a result, cholesterol is implicated in modulating the homeodynamics of a vast majority of cell surface related proteins (Zhang J. et al., 2019). Additionally, cholesterol serves as a crucial component in the formation of microdomains termed lipid rafts, which contain a multitude of proteins (Murai, 2015), that dictate the functional downstream processes such as signaling, proliferation, differentiation, adhesion and apoptosis (Zalba and Ten Hagen, 2017).

**FIGURE 1:**
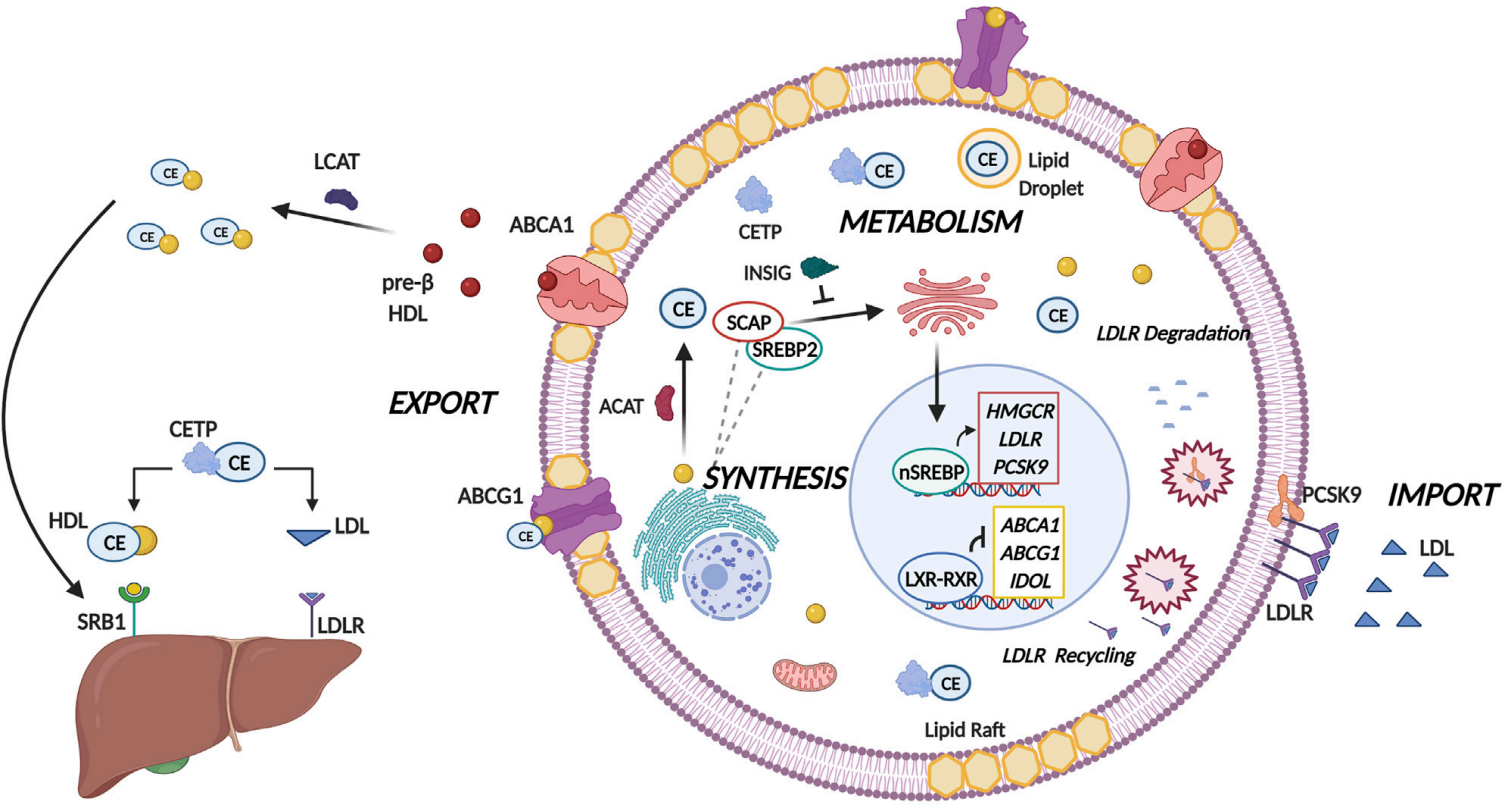
Molecular Mechanisms of Cholesterol Homeostasis. Cholesterol homeostasis is maintained by balancing the levels of cholesterol import, synthesis, metabolism, and export. A pivotal group of transcription factors termed the sterol regulatory element binding proteins (SREBPs) regulate lipogenesis and lipid uptake. When intracellular sterol concentrations are low, cholesterol biosynthesis is induced by SREBP2 where following proteolytic cleavage the nuclear isoform of SREBP2 (nSREBP2) binds to sterol response elements to facilitate expression of lipogenesis target genes namely HMGCR, low density lipoprotein receptor (LDLR) and proprotein convertase subtilisin/kexin type-9 (PCSK-9). HMGCR catalyzes the rate limiting step in cholesterol biosynthesis, whereas the LDLR imports cholesterol from the cellular milieu through endocytosis and hydrolysis of low density lipoprotein (LDL) particles. Collectively, this will lead to increased sterol levels within the cell. PCSK-9 regulates LDLR by either degrading LDLR by recruiting it to lysosome, or by prohibiting the endocytic recycling of LDLR by binding to the epidermal like growth factor repeat of LDLR. To prevent the excess accumulation of cholesterol in the cell, acyl-CoA cholesterol acyltransferase ACAT converts free cholesterol to cholesteryl esters (CEs) which are subsequently transported by cholesteryl ester transfer protein (CETP) from the site of synthesis to sites of storage in lipid droplets. Excess cellular cholesterol and cholesterol-derived oxysterols promotes the Liver-X-Receptor (LXR)-Retinoid-X-Receptor (RXR) mediated transcription of the two principal transporters ATP-binding cassette subfamily A member 1 (ABCA1) and subfamily C member 1 (ABCG1). These transporters transfer cholesterol to pre-β high density lipoprotein (HDL) and HDL respectively where cholesterol can either be eliminated by HDL binding to scavenger receptor- B1 (SR-B1). Alternatively, CETP can mediate the shuttling of cholesterol from HDL to LDL which will then result in elimination of cholesterol by binding to LDLR on the liver.

The synthesis of cholesterol is carried out in a highly conserved manner that is regulated by 21 enzymatic reactions (Silvente-Poirot and Poirot, 2014). In the mitochondria, the tricarboxylic acid (TCA) cycle generates a metabolite termed citrate. Cholesterol synthesis is initiated when citrate is converted to acetyl-coenzyme A (Murai, 2015). Subsequently, through the mevalonate pathway acetyl-coenzyme A is converted to lanosterol in a series of enzymatic reactions that take place in the endoplasmic reticulum (ER) (Murai, 2015). 3-hydroxy-3-methylglutaryl-coenzyme A (HMG-CoA) reductase (HMGCR) is the pivotal enzyme in this process as it catalyses the conversion of HMG-CoA to mevalonate in a rate-limiting step (Murai, 2015). Cholesterol is subsequently formed when the lanosterol is converted to cholesterol either by the Bloch pathway or the Kandutsch-Russell pathway (Murai, 2015). Mammalian cells are known to possess complex cholesterol homeostatic mechanisms (Figure 1) that are regulated at several levels, namely import, synthesis, export, metabolism and esterification (Das et al., 2014). These processes are facilitated by the presence of several important signaling molecules and enzymes at each stage and are summarized in Figure 1. For a more comprehensive overview concerning cholesterol homeostasis, the reader is referred to our previous publication (Gu et al., 2019).

Importantly, cholesterol dyshomeostasis has emerged as a key requirement for cancer initiation and progression (Mandal and Rahman, 2014). Upon carcinogenesis finely tuned mechanisms of cholesterol regulation are altered based on the energetic requirements of the actively dividing cancerous cells (Cruz et al., 2013). Multiple studies conducted validated this notion, highlighting increased expression of sterol regulatory element binding protein (SREBP) target genes including HMGCR responsible for mediating the rate-limiting step in cholesterol synthesis as well as increased production of low density lipoprotein receptor (LDLR) that facilitates cholesterol uptake (Sun et al., 2015; Gallagher et al., 2017; Wen et al., 2018). Interestingly, proprotein convertase subtilisin/kexin type-9 (PCSK-9) (a regulator of LDLR) expression in cancer cells is found to be much lower expressed than their non-cancerous counterparts (Bhat et al., 2015). This allows for the cancer cells to enhance their cholesterol uptake. The decreased expression can be attributed to mutations in PCSK-9 as well as deregulations in cancer signaling pathways (e.g., NF-κβ) that facilitate cholesterol accumulation through decreased PCSK-9 expression (He et al., 2017). In terms of metabolism and efflux, acyl-CoA cholesterol acyltransferase (ACAT) activity in cancer cells has been shown to increase and promotes the conversion of cholesterol to cholesteryl esters (CEs) preventing the accumulation of free cholesterol that is toxic to the cell (Li et al., 2016). As a result of this, cholesterol uptake and synthesis is promoted to meet the metabolic demands of the cancerous cell (Li et al., 2016). Subsequently cholesteryl ester transfer protein (CETP) expression in cancer cells is also increased, which could be explained by the crucial role of CETP in stabilizing the CEs and promoting their storage in lipid droplets (Esau et al., 2016). Contrary to this, LXR modulated genes (such as ABCA1) display decreased expression in multiple cancer types. These deregulations ultimately facilitate accumulation of cholesterol intracellularly, thus contributing to poor prognosis and survival of patients (Kuzu et al., 2016).

From the above it is evident that cholesterol has a multifaceted role in the cell. Cholesterol synthesis is mediated through the highly conserved mevalonate pathway. Mammalian cells are known to possess complex cholesterol homeostatic mechanisms which cancer cells subvert to promote tumor initiation and progression.

### Role of Cholesterol Synthesis in Drug Resistance

Scientific evidence have implicated cholesterol as well as the oxygenated derivatives of cholesterol in the acquisition of drug resistance in cancer. This is facilitated by the influence of cholesterol on cell signaling pathways that contribute to resistance or alternatively through direct effects on the expression and activity of multi-drug transporters.

Kong *et al.*, have documented increased levels of cholesterol due to increased expression of HMGCR in enzalutamide-resistant prostate cancer cell lines relative to enzalutamide-sensitive cell lines, where enzalutamide functions as an androgen receptor inhibitor. Consequently, overexpression of HMGCR in sensitive cell lines can confer resistance whereas shRNA-mediated silencing restores sensitivity (Kong et al., 2018). On this basis, authors documented that treating enzalutamide-resistant cell lines with both simvastatin (a cholesterol synthesis blocker) and enzalutamide efficiently restores sensitivity as evidenced by the decreased half maximal inhibitory concentrations (IC_50_) of enzalutamide in resistant cell lines (Kong et al., 2018). Similarly, administering a membrane cholesterol depletor, methyl-β-cyclodextrin (MβCD) to gefitinib resistant non-small cell lung cancer (NSCLC) cell lines resulted in a significant decrease in IC_50_ of gefitinib as a consequence of inhibition of epidermal growth factor receptor (EGFR) activity as well as the activity of downstream MAPK/ERK and PI3K/Akt signaling pathways (Chen et al., 2018). In lung cancer cells, administration of EGFR inhibitors facilitated synthesis of both cellular and mitochondrial cholesterol (Howell et al., 2020). Following exposure to tyrosine kinase inhibitors (TKIs), drug resistant cells showed an increased expression of 7-dehydrocholesterol reductase, 24-dehydrocholesterol reductase, with an approximate 20-fold increase in the expression of SREBF2. Importantly, the administration of ketoconazole (an inhibitor of CYP51A1) together with a TKI acted synergistically to restore TKI sensitivity in resistant cells (Howell et al., 2020). In another study, administering both simvastatin and EGFR TKIs proved effective in combatting T790M (Thr790Met, gatekeeper mutation) mediated EGFR TKI resistance (Hwang et al., 2014). Inhibiting Akt signaling led to reduced β-catenin phosphorylation and further repression of downstream target genes including cyclin-D1 and survivin, where downregulation of survivin has been found to be crucial in mediating apoptosis induction (Hwang et al., 2014).

Cholesterol biosynthesis genes also contribute to cisplatin-resistance in ovarian cancer. This is evidenced by findings that the expression of SREBP2 dependent-genes, including *HMGCR*, *LDLR* as well as farnesyl-diphosphate farnesyltransferase 1 (*FDFT1*) increase following the administration of cisplatin and is dose-dependent (Zheng et al., 2018). The transition from a sensitive to a resistant phenotype correlates with higher levels of plasma membrane and lysosomal cholesterol in resistant cells (Criscuolo et al., 2020). This suggests that along with blocking the endogenous cholesterol synthesis, treating cells with cholesterol depletors to extract cholesterol from the cellular membranes could possibly combat drug resistance. This would be mediated by increasing the expression of genes involved in cholesterol efflux resulting in a concomitant decrease in intracellular cholesterol content (Yokoo et al., 2015; Zimmer et al., 2016). Therefore, further investigations into the field of employing cholesterol depletion as a means to restore drug sensitivity are required.

### Role of Cholesterol Uptake and Metabolism in Cancer Drug Resistance

Cholesterol uptake due to the increased expression of LDLR facilitated chemoresistance and increased risk of recurrence in pancreatic ductal adenocarcinoma (PDAC) (Guillaumond et al., 2015). Silencing of LDLR led to a disruption in cholesterol distribution, impeding ERK 1/2 signaling, and further sensitized PDAC cells to chemotherapeutic agents both *in-vitro* and *in-vivo* (Guillaumond et al., 2015). Similarly, studies conducted by Naito *et al.* identified the crucial role of LDL in inducing resistance to TKIs in clear cell renal cell carcinoma (ccRCC) as mice fed on a high cholesterol diet (21% milk fat with 1.25% added cholesterol) presented with resistance to sunitinib (TKI) treatment when compared with mice that were on a normal diet, presumably due to LDL mediated activation of the PI3K/Akt signaling pathway (Naito et al., 2017). Consequently, administering a cholesterol depletor, hydroxy-propyl-β-cyclodextrin (HPβCD), resulted in disruption of lipid raft integrity, and abrogated LDL-mediated AKT phosphorylation in the SK-45 RCC cell line as well as the patient-derived PNX0010 ccRCC cell line (Naito et al., 2017).

In addition to LDL, CEs have also been implicated in facilitating chemoresistance. Li *et al.* identified a significant increase in the fraction of lipid droplet area as well as a modest increase in CEs in the G3K (a resistant PDAC cell line) relative to the sensitive Mia-Pa-Ca2 PDAC cell line (Li et al., 2018). Consequently, administration of an ACAT1 inhibitor avasimibe proved effective in reducing cell viability of both Mia PaCa-2 and G3K cells, whereas administering both avasimibe and gemcitabine enhanced the anti-proliferative effect relative to monotherapy (Li et al., 2018). Authors documented synergism between avasimibe and gemcitabine, and further *in-vivo* analysis identified that treatment with only gemcitabine resulted in tumor relapse after 20 days, whereas a combination of gemcitabine and avasimibe effectively reduced tumor growth with no observable relapse following 34-days of treatment (Li J. et al., 2018). The combination therapy proved most effective in reducing the levels of CEs as well as the expression of *p*-AKT highlighting that avasimibe restores chemosensitivity in gemcitabine-resistant cells by impeding Akt signaling (Li et al., 2018). Attempting to demonstrate the link between cholesterol and gallbladder cancer, Zhang and colleagues established that gallbladder cancer patients present with increased expression of genes involved in cholesterol biosynthesis (*HMGCR*) as well as sterol sulfonation (*SULT2B1*), reduced expression of genes *CYP7B1* and *CYP39A1* responsible for cholesterol catabolism as well as decreased expression of genes that facilitate cholesterol efflux (*ABCA1, ABCG5, LCAT and CETP*) (Zhang Y. et al., 2019). Treating gallbladder cancer cells with both lovastatin and cisplatin proved effective in increasing apoptosis in cells when compared with single treatments. Interestingly cholesterol depletion abrogated activation of checkpoint kinase (CHK) 1, CHK2 as well as γ-H2AX following treatment with cisplatin highlighting reduced DNA damage response (Zhang Y. et al., 2019). This effect was also observed *in-vivo* indicating reduced growth in the combination-treatment groups (Zhang Y. et al., 2019). Furthermore, Esau *et al.* by employing both *in-vitro* and *in-vivo* assays identified that administration of Acetyl Plumbagin (AP) leads to a decrease in the mRNA expression and protein levels of CETP which results in growth inhibition and contributes to mitochondrial-mediated apoptosis (Esau et al., 2016). On this basis, silencing CETP in MCF-7 cells is shown to enhance the sensitivity of cells to Tamoxifen (TAM) as well as AP as evidenced by the increased caspase 3/7 activity and increased percentage of cells undergoing apoptosis when compared to the non-transfected cells (Esau et al., 2016). Studies conducted by Gu *et al.* further identify that administering a combination treatment of TAM and AP in CETP knockout MCF-7 cells functions synergistically to enhance mitochondrial-mediated apoptosis relative to monotherapy with either TAM or AP (Gu et al., 2019).

### Effect of Cholesterol on Multidrug Resistant Transporters

Further developments in this field are beginning to reveal the crucial role cholesterol plays in regulating the expression as well as activity of principal multi-drug transporters contributing to MDR. This is elucidated by the observation that cholesterol serves as a transcriptional regulator of MDR efflux transporters where a positive correlation between LXRα/β protein expression and MDR1 expression has been documented. (Kim S. et al., 2018). In terms of MDR transporter activity, plasma membrane cholesterol content alters membrane fluidity thereby regulating their activity (Kopecka et al., 2020). Interestingly, the lipid environment surrounding these transporters modulate their conformation, maintaining transporters in a low-energy stable conformation (Kopecka et al., 2020). With respect to cholesterol, this molecule surrounds P-gp adopting an asymmetrical distribution together with phospholipids (Alam et al., 2019). The outer leaflet is characterized by an ordered ring of cholesterol whereas the inner leaflet presents with both phospholipids and cholesterol. Importantly, the lipid-transporter interactions mediate structural modifications of P-gp consequently regulating its catalytic activity (Alam et al., 2019). Additionally, studies conducted document that P-gp substrates accumulate in cholesterol-rich regions of the membrane thereby enhancing the activity of the P-gp (Subramanian et al., 2016). Interestingly, evidence exists highlighting the role of cholesterol in altering P-gp substrate K_M_ values thereby implying that cholesterol directly interacts with substrate binding sites (Hegedüs et al., 2015). This is illustrated mechanistically, by studies conducted by Clouser *et al.* proposing that plasma membrane cholesterol modulates P-gp-transmembrane helix-membrane interactions which consequently promotes long-range conformational changes in the nucleotide-binding domain of P-gp promoting a drastic increase in the rate of ATP hydrolysis (Clouser et al., 2021). This consequently justifies the crucial role cholesterol plays in supporting the activity of P-gp housed within lipid rafts.

The acquisition of chemoresistance following platinum-based therapy is regulated by cholesterol-mediated influence on the expression of ABCG2 (Wu et al., 2015). In this study, individuals who displayed quick chemoresistance presented with increased levels of baseline total cholesterol in tumour tissue relative to the delayed chemoresistance group. Additionally, mRNA and protein expression analysis documented a significant increase in the levels of ABCG2 (Wu et al., 2015). Further, *in-vitro* studies have shown that culturing the A549 lung cancer cell line in cell culture media supplemented with high cholesterol significantly increased the IC_50_ of platinum-based chemotherapeutic agents, whereas treating cells with an ABCG2 blocker, Nicardipine or pravastatin combats cholesterol-induced chemoresistance (Wu et al., 2015). Similarly, pre-treatment with cholesterol (cholesterol repletion) resulted in a significant increase in the IC_50_ to cisplatin and paclitaxel in PA-1 and SCOV-3 ovarian cancer cells which is attributed to cholesterol-mediated increase in the expression of ABCG2 and MDR1 (Kim S. et al., 2018). Interestingly, a positive correlation between LXRα/β protein expression and MDR1 expression has been documented and siRNA-mediated silencing of LXRα/β abrogated cholesterol-mediated induction of MDR1 consequently affecting cholesterol-induced chemoresistance (Kim S. et al., 2018).

Targeting cellular cholesterol is seen to affect lipid raft integrity consequently abolishing the activity of multidrug transporters leading to enhanced retention of chemotherapeutic agents. The treatment with lovastatin and imatinib enhanced cytotoxicity of imatinib in cell lines as well as CD34^+^ chronic myelogenous leukaemia (CML) cells derived from patients diagnosed with different disease stages (Glodkowska-Mrowka et al., 2014). This is attributed to the inhibition of ABC transporter activity namely ABCB1 and ABCG2 leading to increased retention of imatinib in CML cells (Glodkowska-Mrowka et al., 2014). It is important to note that the expression of these transporters is not affected following treatment with lovastatin. By impeding the activity of these transporters, the accumulation of imatinib in CML cells is enhanced thereby allowing for increased amounts of the active drug to bind to and exert antileukemic activity (Glodkowska-Mrowka et al., 2014). In terms of pancreatic cancer, the principal CD133 marker known to co-localize to lipid rafts display a tendency to co-occur with mevalonate synthesis pathway enzymes (Gupta et al., 2018). Importantly, attempting to eradicate this resistant cancer cell population by the administration of lovastatin alone does not significantly affect cell viability in CD133 + cells, however co-administration of lovastatin and paclitaxel induces cell death in CD133 + cells by disrupting lipid raft integrity which consequently abrogates ABC transporter activity (Gupta et al., 2018).

The above summarised studies emphasize the importance of cholesterol in driving cancer proliferation and therapeutic response by affecting signaling pathways that facilitate resistance or through the direct effects on the expression and activity of MDR transporters. Emerging scientific evidence document that targeting cholesterol through gene silencing, cholesterol synthesis inhibition or cholesterol depletion restores sensitivity to conventional chemotherapeutics.

Seeing that drug resistance is a hallmark of metastatic cancer, there is a gap present in the literature regarding the role of cholesterol in cells undergoing the tumor cell plasticity program of Epithelial-Mesenchymal Transition (EMT). In the following sections, we have addressed this gap at least partly by reviewing the published literature and proposing a potential hypothesis to demonstrate the crucial involvement of cholesterol in EMT and metastasis.

### Epithelial-Mesenchymal Transition (EMT) and Its Regulation

**FIGURE 2:**
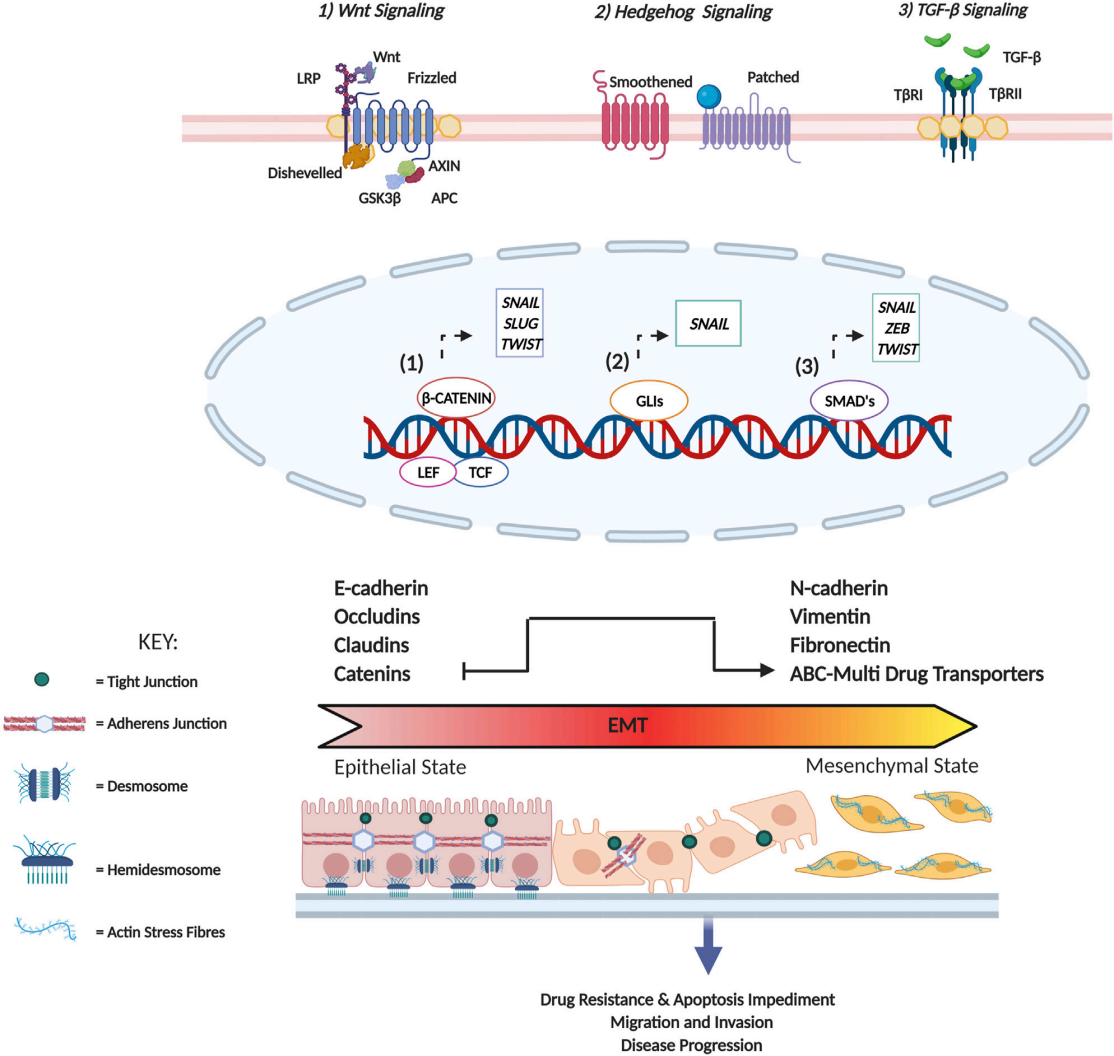
Summary of EMT induced changes in cellular physiology. In tissues, epithelial cells are often arranged as either a single layer or exist as multi-layered sheets. These cells are characterized by the presence of specialized contacts called cell junctions that facilitate cell-cell interaction (cadherin-based adherens junctions, tight junctions, and desmosomes) as well as cell-matrix interactions (hemidesmosomes) that maintain structural integrity and apical-basal polarity. In terms of cytoskeletal composition, Type I intermediate filaments such as cytokeratins are expressed, and adherens junctions serve to indirectly connect cortical actin filaments of two adjacent cells. On the contrary, mesenchymal cells are characterized by minimal cell junctions and detached stromal arrangements promoting front-rear polarity. In terms of cytoskeletal composition, vimentin is predominantly expressed to enhance cytoskeletal strength contributing to cell flexibility, and actin fibres arrange to form stress fibres. Additionally, the formation of actin-rich membrane projections facilitates cellular movement as well as sensory reception. Characteristic surface markers utilized to identify epithelial cells include E-cadherin, cytokeratins, occludins as well as claudins, while mesenchymal cells are identified based on the presence of N-cadherin, fibronectin, and vimentin. Cellular stimuli lead to the activation of several signaling pathways (Wnt, Sonic Hedgehog and TGF-β) that promote the expression of master EMT TFs (SNAIL, SLUG, TWIST and ZEB). Following activation of the EMT program these TFs facilitate the loss of several of the aforementioned epithelial characteristics, promote cytoskeletal remodeling and initiate a concomitant gain of mesenchymal traits. This transition consequently facilitates a change in cell morphology from squamous, cuboidal, columnar to a fibroblast/spindle-like morphology conferring upon cancer cells migratory and invasive potential.

EMT is classified as a tumor cell plasticity program. Initially known to play a role in embryonic development, current scientific evidence supports the role of EMT in various pathological processes including wound healing, fibrosis, inflammation and cancer (Yeung and Yang, 2017). The outline of the EMT program is summarised in Figure 2.

Cells exhibit a significant degree of plasticity where instead of transitioning between full epithelial to full mesenchymal phenotype, they traverse through a spectrum of intermediate states along the epithelial-mesenchymal axis expressing varying levels of epithelial and mesenchymal markers (Nieto et al., 2016).

Repression of the epithelial phenotype and activation of the mesenchymal phenotype is dictated by regulatory networks that function to modulate the activity of a group of master transcription factors (TFs) (Fuxe et al., 2010; Craene and Berx, 2013). The master TFs consist of the zinc-finger E-box-binding homeobox factors ZEB1/2 and SNAIL 1/2 as well as the basic helix-loop-helix (bHLH) factors TWIST 1/2 which are also the most comprehensively studied (Nieto et al., 2016). Transcriptional and translational controls of these TFs are governed by the expression of specific non-coding RNAs including microRNAs (miRNAs) and long non-coding RNAs (lncRNAs) as well as alternative splicing and protein stability (Craene and Berx, 2013; Nieto et al., 2016). Interestingly, the master EMT-TFs function co-operatively to modulate the expression of target genes as well as each other (Shibue and Weinberg, 2017; Dongre and Weinberg, 2019). In this way, the reciprocal interactions induce comprehensive EMT associated changes even in the event where only one of the TFs are activated (Shibue and Weinberg, 2017). The role of each of these master TFs in facilitating EMT induction is briefly elaborated on below (also see Figure 2).

#### SNAIL Family of Transcription Factors

The SNAIL subfamily of zinc-finger TFs is represented by three members in vertebrates namely Snail (Snail 1), Slug (Snail 2) and Smuc (Snail 3). The transcriptional repression of E-cadherin is achieved through direct binding of Snail and Slug to E-box regions in the proximal promoter region (Batlle et al., 2000; Hajra et al., 2002), while emerging scientific evidence continue to illustrate the recruitment of epigenetic regulators that affect both DNA and chromatin accessibility, and as such E-cadherin expression (Peinado et al., 2004; Herranz et al., 2008; Hou et al., 2008; Lin et al., 2010; Dong et al., 2012; Tong et al., 2012; Wu et al., 2012). Additionally, Snail and Slug are also implicated in repressing the expression of occludins, claudins and CXAR (CAR) which are present in tight junctions, crucial to maintaining cell polarity (Ikenouchi et al., 2003; Martínez-Estrada et al., 2006; Wang et al., 2007; Vincent et al., 2009). Conversely, Snail and Slug can upregulate key mesenchymal markers namely vimentin and fibronectin (Cano et al., 2000; Jethwa et al., 2008).

#### Basic helix-Loop-Helix Transcription Factors

While several members of this family have been identified, TWIST1 has been documented to play a pivotal role in EMT by repressing the expression of epithelial proteins and by promoting the expression of mesenchymal related proteins. Twist1 binds to E-cadherin promoter regions, recruiting epigenetic regulators that influence DNA and chromatin accessibility as well as modulating expression of ncRNAs that play a role in EMT-induction (Yang et al., 2004; Fu et al., 2011; Yang et al., 2012; Meng et al., 2018).

#### Zinc-Finger E-Box Binding Homeobox (ZEB) Family

The ZEB family of TFs in humans comprises ZEB1 and ZEB2 and are classified as zinc finger/homeodomain TFs (Abba et al., 2016; Georgakopoulos-Soares et al., 2020). These TFs possess characteristic protein-binding domains which facilitate the recruitment of co-repressors as well as co-activators leading to gene repression or gene activation, respectively (Zhang et al., 2015). Based on this dual function, they can induce EMT by employing both repression of epithelial proteins and activation of mesenchymal proteins (Bindels et al., 2006).

Interestingly, completion of the EMT program resulting in cells adopting an extreme mesenchymal phenotype has been noted as an infrequent event in both neoplastic and non-neoplastic human tissue (Derynck and Weinberg, 2019). Predominantly, the stable existence of cells in the hybrid state has been linked with increased metastatic potential since the hybrid EMT state facilitates collective cell migration and reinforces ECM attachment (Saitoh, 2018). Furthermore, the plasticity associated with the hybrid phenotype facilitates the acquisition of stemness as well as drug resistance and correlates with poor survival in several cancer types (Pastushenko and Blanpain, 2019; Liao and Yang, 2020). Yet the precise molecular mechanisms governing the process of EMT-mediated stemness is lacking (Dongre and Weinberg, 2019). Additionally, future studies aimed at identifying whether existence in different hybrid states confer differential response to chemotherapeutic agents would be highly informative to elucidate mechanisms governing drug resistance (Thankamony et al., 2020).

Principally, the induction of EMT is associated with an increase in drug resistance potential in cancer cells. This is seen in response to treatment with platinum-based agents, TKI’s as well as taxane-based cancer therapeutics. To sustain resistance to chemotherapeutic agents, cancer cells continuously evolve. In terms of EMT and its link to drug resistance, cancer cells activate several EMT-related signaling pathways that concomitantly induce the expression of EMT master TFs. These TFs consequently regulate key proteins involved in cell survival and apoptosis signaling. This evolutionary process facilitates the acquisition of survival mechanisms that allow for a certain degree of genetic stability to be maintained in the pursuance of cancer cell proliferation and apoptosis induction impediment following DNA damage. Another mechanism employed by EMT to mediate drug resistance is through the presence of several binding sites in the promoter regions of the ATP-binding cassette (ABC) transporters leading to overexpression, which in turn mediates excessive drug efflux. Since altered cellular metabolism has been linked with EMT, we attempt to discuss the emerging role that cholesterol plays in regulating critical signaling pathways associated with EMT. We further elaborate on this concept by discussing hypoxia as an extrinsic factor that modulates cholesterol to mediate EMT-induced drug resistance.

### Cholesterol Regulates Key Signal Transduction Pathways of EMT Induction and Maintenance to Potentiate Disease Aggressiveness and Resistance

**FIGURE 3:**
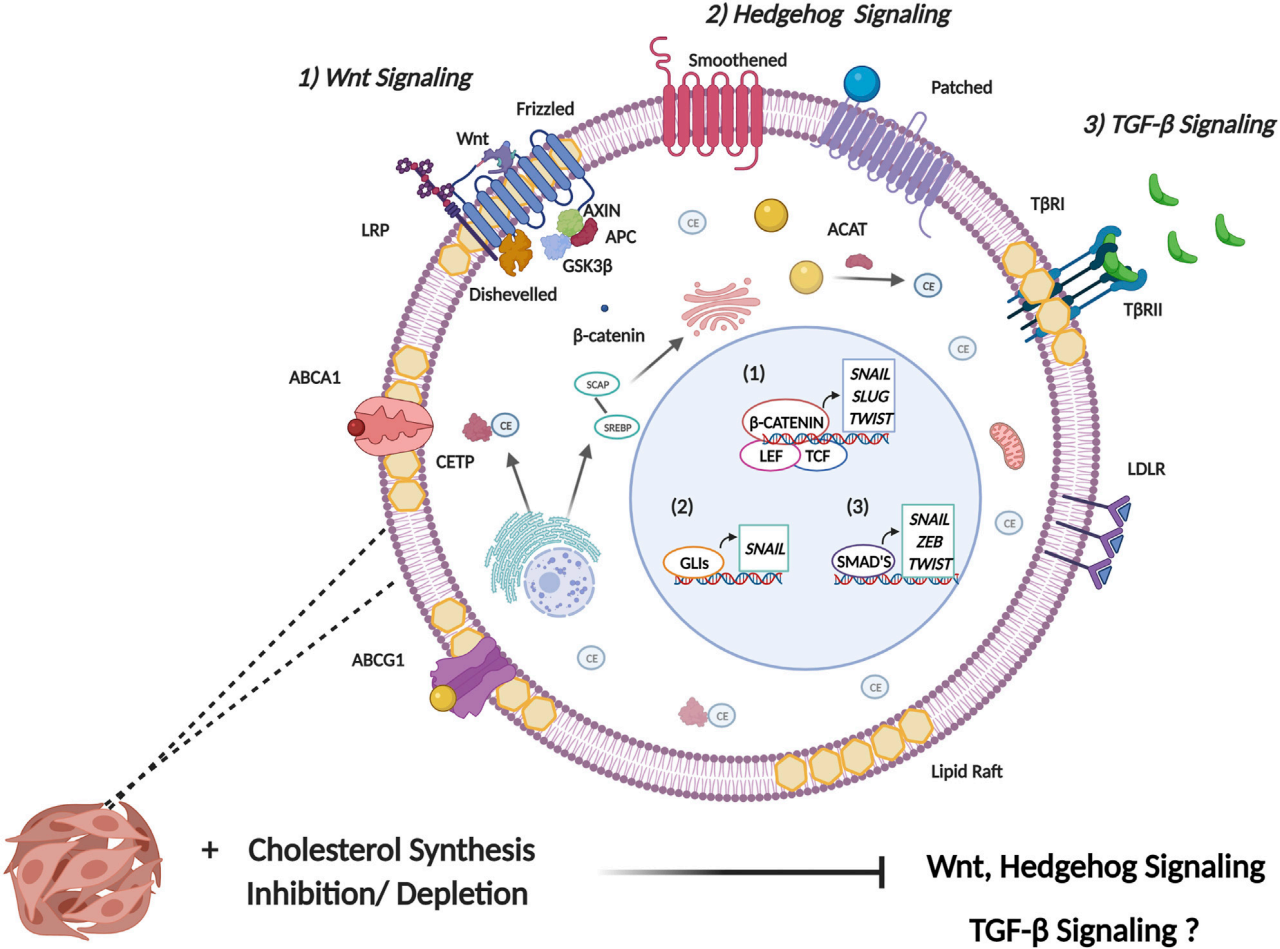
Cholesterol modulates signaling pathways involved in EMT induction. EMT is induced by several signaling pathways that facilitate the expression of EMT master transcription factors (SNAIL, SLUG, ZEB, and TWIST). 1) Canonical Wnt signaling is initiated following the binding of a Wnt ligand to Frizzled receptor. This consequently mediates the release of β-catenin from the glycogen synthase kinase 3-β (GSK3β), axis inhibition protein (AXIN), adenomatous polyposis coli (APC) protein destruction complex. This allows for increased stability of β-catenin where it enters the nucleus binding to the transcription factors T-cell factor (TCF) and lymphoid-enhancer factor (LEF) to induce transcription of EMT TFs. Primarily, Wnt binds to receptors in lipid raft domains and cholesterol facilitates recruitment of the Dishevelled to the plasma membrane. Collectively this facilitates activation of the canonical Wnt/β-catenin signaling. 2) Activation of canonical hedgehog signaling occurs through binding of the hedgehog ligand, sonic hedgehog (Shh) to the transmembrane receptor patched which leads to de-repression of Smoothened. Smoothened consequently activates the transcriptional activity of the GLI family of transcription factors. Cholesterol is necessary for post-translational modification of Hh ligands. Furthermore, the activity of the membrane transducer Smo is regulated directly by cholesterol through its cysteine-rich domain and also indirectly through Hh receptor PTCH1 which possesses a sterol-sensing domain. 3) Transmembrane signaling by TGF-β is initiated upon ligand binding to membrane bound serine/threonine kinase receptor, termed TGF-β type II receptor (TβR-II). This leads to recruitment of membrane bound TGF-β type I receptor (TβR-I) resulting in the formation of a heterotetrameric complex. The main mechanism of intracellular TGF-β signaling is mediated through the Smad family of transcription factors which serve to activate or repress TGFβ responsive genes. The role of cholesterol in modulating TGF-β signaling is complex and not fully understood, with some studies implicating cholesterol in TGF-β signaling, and other studies highlighting reduced cholesterol upon TGF-β induced EMT.

The cellular EMT process is a complex labyrinth dependent on subversion of critical cellular signaling pathways, which crosstalk extensively to mediate the initiation and regulation of the EMT program (Fuxe et al., 2010). Based on the pleotropic role of cholesterol in the cell, it is not surprising that cancer cells have evolved several mechanisms to facilitate cholesterol dyshomeostasis, which in turn influence several EMT associated signaling pathways. In addition to meeting the increased metabolic demands of cancer cells, deregulated cholesterol metabolism also facilitates increased cellular cholesterol availability which is crucial to regulating the activity of protein intermediates in EMT-related signaling pathways. Furthermore, an increase in cholesterol content corresponds to an increase in lipid raft presence. Since lipid rafts contain several cell signaling proteins, alteration to lipid raft domains may therefore influence cell signaling pathways that ultimately augment neoplastic transformation (Zalba and Ten Hagen, 2017). These rafts in turn regulate crucial processes that cancer cells subvert such as survival, proliferation, migration, invasion, and apoptosis. Intra- or extracellular stimuli mediate alterations in lipid raft size and protein composition facilitating receptor-ligand interactions as well as interactions between proteins leading to downstream activation of signal transduction cascades crucial for cancer cell signaling (Mollinedo and Gajate, 2015). The role of cholesterol in regulating some of the most well studied EMT-related signaling pathways (Figure 3) will be further delineated below. It should be noted that this section purely aims to elucidate the role cholesterol plays in the regulation of these EMT-related pathways. The reader is suggested to refer to several existing reviews (Lamouille et al., 2014; Singh et al., 2018; Das et al., 2019; Dongre and Weinberg, 2019) that excellently delineate the precise mechanisms these pathways employ to induce EMT.

#### Wnt Signalling Pathway

In canonical Wnt signalling, binding of Wnt ligands to Frizzled (Fz) receptors in conjunction with low-density lipoprotein receptor-related protein (LRP) facilitates recruitment of Dishevelled (Dvl) protein to the plasma membrane (Zhong and Virshup, 2020). This recruitment is facilitated by cholesterol (Sheng et al., 2014), which in turn supports activation of canonical/β-catenin dependent Wnt signaling (Sezgin et al., 2017). Additionally, Wnt binding to the receptor complex occurs exclusively in the plasma membrane ordered compartments termed lipid rafts (Sezgin et al., 2017). Consequently, administering enzymes/drugs that disrupt key constituents in ordered plasma membrane significantly decreased Wnt/β-catenin signaling when cholesterol was disrupted following the administration of cholesterol oxidase (Sezgin et al., 2017). Furthermore, a class of cholesterol depleting agents termed cyclodextrins have recently been implicated in modulating Wnt signaling through cholesterol depletion from membrane lipid rafts. Lipid rafts play a pivotal role in maintaining the integrity of LRP6 in triple negative breast cancer (TNBC). Treating TNBC cell lines with MβCD resulted in reduced proliferative and migratory potential and also increased apoptosis induction by downregulating several anti-apoptotic proteins as well as decreased expression of LRP6 and β-catenin (Badana et al., 2018). This suggests that lipid raft integrity is crucial in dictating the functionality of the Wnt/β catenin signaling which plays a key role in facilitating TNBC aggressiveness.

#### Sonic Hedgehog Signalling

Sonic hedgehog (Shh) signaling is essential for embryonic development but has also been documented to play a pivotal role in tumorigenesis and cancer stem cell maintenance (Wu et al., 2017). Hedgehog (Hh) signal transduction can be linked with cholesterol through the post-translational modification on the Hh ligands where covalent modification is initiated at the c-terminal end of the ligand through cholesterolysis (Callahan and Wang, 2015; Blassberg and Jacob, 2017). The translocation of cholesterolysis-defective Hh precursors is impeded from the ER to the Golgi. As they are sequestered in the ER, ER associated protein degradation (ERAD) mediated polyubiquitination and proteasomal degradation is induced which abrogates translocation of the protein out of the ER and hence Hh signaling (Hampton, 2002). In addition to this, the Hh receptor PTCH1 is also documented to possess a sterol-sensing domain (SSD) which is typically present in proteins regulating cholesterol synthesis and transport (Luchetti et al., 2016). A study conducted by Bidet *et al.* proposes that the SSD of PTCH1 allows for cholesterol efflux from the cell when a Shh ligand is absent, this consequently results in a decrease in the intracellular concentration of cholesterol and impedes the activity of the membrane transducer Smo (Bidet et al., 2011). Conversely, binding of a ligand to PTCH1 facilitates the uptake of PTCH1 and impedes the efflux of cholesterol. The increased intracellular concentration of cholesterol mediates increased abundance of Smo at the membrane (Bidet et al., 2011). Importantly, cholesterol is also seen to directly regulate the activity of Smo by binding to its extracellular cysteine-rich domain (CRD). Mutations found in the CRD impedes cholesterol mediated Smo activation and consequently Shh mediated signaling hence indicating the important role of cholesterol in endogenous Hh signaling (Luchetti et al., 2016).

#### Transforming Growth Factor—β (TGF-β) Signalling

TGF-β is a pleiotropic cytokine known to play an important role during embryonic development as well as regulate homeostasis of adult tissues by modulating processes such as cell proliferation, differentiation, migration, invasion, immunological response and apoptosis (Chruścik et al., 2018; Ahmadi et al., 2019; Derynck and Budi, 2019; Yang et al., 2020). Consequently, aberrant TGF-β signaling often leads to dedifferentiation of several cell types, immunosuppressive effects, enhanced angiogenesis and accommodates an inflammatory milieu which consequently contributes to tumorigenesis (Padua and Massagué, 2009).

The formation of TβRI/TβRII/TGβ-1 complex is dependent on lipid raft integrity and disruption of lipid rafts impede canonical TGF-β signaling (Ma et al., 2007). Importantly, the localization of TGF-β receptors in lipid rafts is crucial to mediating TGF-β induced activation of MAPK signaling and promoting EMT and cell migration. Contrary to the afore-mentioned studies, a study conducted by Zhao *et al.* suggests that down-regulation of cholesterol may be a consequence of EMT (Zhao et al., 2019). It was recently identified that TGF-β promotes the enrichment of ZEB1 and CtBP at the promoter region of SREBF2 leading to repression of SREBF2 and its downstream target genes. Interestingly the reduction in membrane-bound cholesterol was implicated in enhancing the stability of TGFβRI (Zhao et al., 2019). Additionally, studies conducted by Shapira *et al.* elucidated that cholesterol depletion facilitates a PKR-dependent phosphorylation of eIF2α which enhances the translation of c-Jun (Shapira et al., 2018). An increase in JNK-mediated c-Jun phosphorylation is also reported which enhances the activation of c-Jun and consequently increases the transcription of Smad 2/3. This ultimately allows for enhanced TGF-β mediated signaling (Shapira et al., 2018). These studies elucidate the complex role that cholesterol plays in regulating the expression and activity of TGF-β signaling module. This consequently necessitates additional investigations into the role of cholesterol in the regulation of TGF-β signaling in a cancer setting to identify whether targeting cellular cholesterol is a feasible means to halt cancer progression.

Based on the principal role of cholesterol in regulating key pathways associated with EMT induction and maintenance, emerging scientific evidence now exist implicating cholesterol-related genes in mediating EMT consequently potentiating an aggressive disease phenotype with increased drug resistance. Studies describing the role of cholesterol-related genes in EMT induction, metastasis and drug resistance are summarized in Table 1 below.

**TABLE 1:** Summary of cholesterol related genes and their link to EMT, metastasis and drug resistance.

Gene symbol	Description	Mode of action	Role in EMT, metastasis and drug resistance	References
*ABCA1*	ATP Binding Cassette Transporter Subfamily A Member 1	Cholesterol efflux pump/cholesterol transport/reverse cholesterol transport/cholesterol homeostasis	ABCA1 overexpression in CRC facilitates the induction of EMT leading to increased migratory and invasive potential by regulating the stability of Cav-1	Aguirre-Portolés et al. (2018)
			Overexpression of ABCA1 significantly impeded eicosapentaenoic acid and doxorubicin therapy-induced cell polarisation and reduced apoptosis induction	Torres-Adorno et al. (2019)
			Overexpression of ABCA1 in human breast cancer mediates cell migration by modulating cellular cholesterol levels and is associated with increased metastasis	Zhao et al. (2016)
			High expression levels of ABCA1 enhances ovarian cancer cell growth and migration which is attenuated following statin administration	Hedditch et al. (2014)
			HDL induces increased proliferation and migration in androgen independent prostate cancer cell lines employing an ABCA1 dependent mechanism	Sekine et al. (2010)
*ABCG1*	ATP Binding Cassette Transporter Subfamily G Member 1	Macrophage cholesterol and phospholipids transport/cholesterol transport/cholesterol homeostasis	Confers chemoresistance in lung adenocarcinoma	Zhan et al. (2019)
*DHCR7*	7-Dehydrocholesterol reductase	Cholesterol Biosynthesis/catalyses the conversion of 7-dehydrocholesterol to cholesterol	--------	
*LRP10*	LDL Receptor Related Protein 10	Uptake of apolipoprotein E-containing lipoproteins	--------	
*LRP1B*	LDL Receptor Related Protein 1B	Wide variety of roles in normal cell function and development due to their interactions with multiple ligands	Deletion/Downregulation of LRP1B displays a significant correlation with acquired resistance to liposomal doxorubicin in high grade serous ovarian cancer	Cowin et al. (2012)
			siRNA-induced silencing of LRP1B enhances migration and metastasis of colon cancer cells and upregulates the expression of N-cadherin and Snail	Wang et al. (2017)
*OLR1*	Oxidized Low Density Lipoprotein Receptor 1	OLR1 gene encodes the LOX-1 receptor protein which internalizes and degrades oxidized low-density lipoprotein	Overexpression of LOX-1 in prostate cancer cells which when activated by oxLDL leads to Snail and Slug mediated EMT induction. This promotes actin cytoskeleton restructuring and activates MMP2 and MMP9 facilitating cancer cell migration and invasion	González-Chavarría et al. (2018)
			TNF-α mediated upregulation of endothelial cell LOX-1 mediates adhesion and *trans*-endothelial migration of MDA-MB-231 cells	Liang et al. (2007)
			Overexpression of OLR1 enhanced osteosarcoma cell proliferation, and mediated EMT-induced cell migration and invasion which consequently promotes the formation of lung metastases *in-vivo*	Jiang et al. (2019)
			LOX-1 induced activation of the PI3K/Akt/GSK3β pathway facilitates EMT induction in gastric cancer cells and enhances migratory and invasive potential. Consequently, overexpression of LOX-1 in gastric cancer tissue correlates with a poor prognosis	Li et al. (2017)
			OxLDL by binding to the LOX-1 receptor facilitated activation of the NFκβ pathway promoting the upregulation of VEGF-C expression in gastric cancer cells. This consequently promotes lymphangiogenesis and lymphatic metastasis of gastric cancer cells	Ma et al. (2019)
			OLR1 promoted proliferation and EMT-mediated migration and invasion *in-vitro* and metastasis of pancreatic cells *in-vivo* which is facilitated by *c-myc* induced activation of HMGA2 transcription	Yang et al. (2020)
			LOX-1 overexpression induces EMT in pancreatic cancer cell lines which may facilitate enhanced migratory and invasive potential	Zhang et al. (2018)
*OSBPL1A*	Oxysterol Binding Protein Like 1A	Cholesterol metabolism	------	
*PCSK-9*	Proprotein convertase Subtilisin/Kexin Type 9	LDL receptor associated proteins/cholesterol homeostasis	PCSK-9 deficiency abrogates liver metastasis by decreasing cholesterol levels	Sun et al. (2012)
*PRKAG2*	Protein kinase AMP-Activated Non-Catalytic Subunit Gamma 2	Regulating *de novo* biosynthesis of fatty acid and cholesterol	------	
*SORL1*	Sortilin Related Receptor 1	LDL associated proteins/cholesterol metabolism	------	

Based on the above documented literature, it can be stated that cholesterol dyshomeostasis influences several EMT associated signaling pathways including Wnt, Shh and TGF-β. This is achieved by directly regulating the activity of EMT-related protein intermediates or through cholesterol’s lipid raft modulating properties. While increased cholesterol is seen to facilitate Wnt and Shh signaling, cholesterol plays a more complex role in the regulation of TGF-β signaling.

Recent scientific evidence also highlight the important role played by the tumor microenvironment in facilitating EMT and drug resistance. The following section of the review aims to discuss the significant contribution of hypoxia to EMT-mediated drug resistance. This is achieved through the direct effect of hypoxia on multidrug transporters and apoptosis impediment as well as indirectly through its immunomodulatory function in the tumour microenvironment. Furthermore, we will also attempt to shed light on mechanisms delineating the effect of hypoxia on cellular cholesterol levels and the resulting effect this has on EMT-mediated drug resistance. This will be completed with the intention of elucidating whether targeting cellular cholesterol could be employed as an efficient novel therapeutic means to combat EMT-mediated drug resistance.

### Hypoxia and its Link to EMT-Mediated Drug Resistance

**FIGURE 4:**
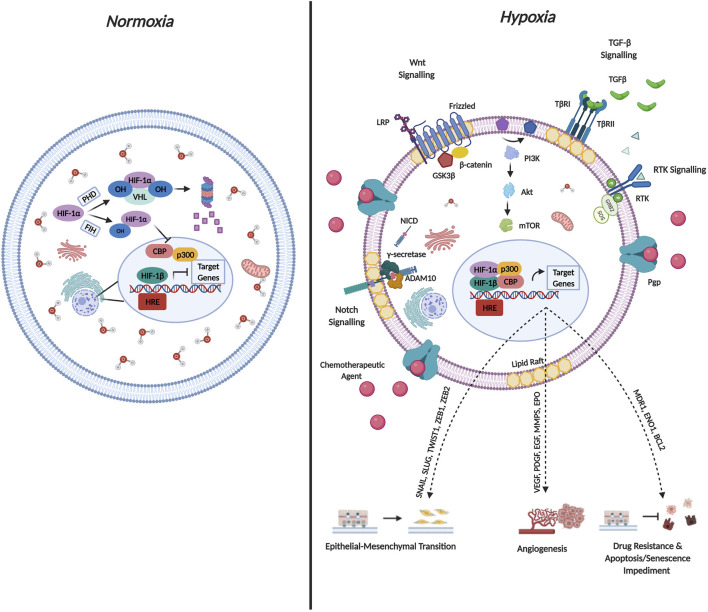
Molecular Mechanisms Governing HIF-1α Regulation and Cellular Function. Hypoxia-inducible factors (HIF’s) occur as heterodimers containing an oxygen sensitive subunit (α) and a constitutively expressed β subunit. Both subunits possess a basic helix-loop-helix (bHLH) as well as PER/ARNT/SIM (PAS) domain that is required for DNA binding and dimerization. The oxygen dependent degradation domain (ODD) of HIF-1α mediates oxygen dependent degradation. Under normoxic conditions, prolyl hydroxylase enzymes, hydroxylate HIF-1α on conserved Pro residues within the ODD facilitating von-Hippel-Lindau mediated ubiquitination and subsequent proteasomal degradation. Additionally, Asn-hydroxylation mediated by factor-inhibiting HIF-1α impedes interaction with the co-activators p300/CBP co-activator. Both mechanisms are documented to abrogate HIF transcriptional activation under normoxic conditions. Under conditions of hypoxia, the hydroxylases lose their activity, leading to HIF-1α stabilization. HIF-1α subsequently translocates to the nucleus and binds to HIF-1β as well as the p300/CBP co-activator forming a complex. The activated complex binds to multiple hypoxia-responsive elements (HREs) in the promoter regions to activate transcription of target genes. The concomitant expression of target genes mediates cancer aggressiveness through EMT and angiogenesis induction as well as drug resistance and apoptosis impediment.

Cancer cells have evolved several mechanisms to synthesize new blood vessels from pre-existing vessels through angiogenesis, and their vasculature often display abnormal characteristics such as distended capillaries, leaky walls, and irregular blood flow (Nurwidya et al., 2012). Additionally, as the cancer progresses, the rate of cell proliferation exceeds the rate of neo-angiogenesis leading to a mismatch between the supply of oxygen and its consumption by the tumor (Challapalli et al., 2017). This in turn results in decreased oxygen levels (≤2%) leading to a condition termed hypoxia (Chee et al., 2019). Hypoxia has been implicated in altering the behavior of cancer cells by manipulating various oxygen-sensitive pathways. The most comprehensively studied, is mediated by the hypoxia-inducible factor (HIF) family of TFs (Nurwidya et al., 2012). Figure 4 briefly describes the molecular mechanisms governing HIF-1α mediated regulation during both normoxic and hypoxic conditions and the resulting changes in cellular physiology that contribute to cancer cell aggressiveness.

In particular, HIF-1α is implicated in contributing to EMT based on its ability to increase the expression of EMT drivers namely SNAIL, ZEB1 and TWIST. Additionally, various signaling pathways described above are activated by hypoxia which are critical to EMT induction including the Notch, Wnt/β-catenin, Sonic Hedgehog as well as the TGF-β pathways (Tirpe et al., 2019). Furthermore, hypoxic conditions increase the production of EMT-induced inflammatory cytokines such as tumor-necrosis factor α (TNF-α), interleukin-6 (IL-6) as well as IL-1β (Bao et al., 2012). Interestingly, hypoxia also contributes to the acquisition of the EMT phenotype by regulating DNA methylation, histone modification as well as the expression of non-coding RNAs required for EMT induction (Choudhry and Harris, 2018).

In terms of hypoxia and drug resistance, early studies conducted by Comerford *et al.* identified that exposing cells to hypoxic conditions increased the mRNA expression of MDR1 by approximately 7-fold and the MDR1 gene harbored a binding site for HIF-1α (Comerford et al., 2002). Chen *et al.* documented that both HIF-1α and MDR1 are upregulated following hypoxic exposure (Chen et al., 2014). Interestingly, TNBC cell lines treated with either paclitaxel or gemcitabine demonstrated an increase in the levels of reactive oxygen species which consequently led to the induction of HIF-1α and HIF-2α, and subsequent transcriptional activity (Samanta et al., 2014). Accordingly, co-administration of a HIF-1 inhibitors (digoxin or acriflavine) proved effective in combatting resistance to paclitaxel/gemcitabine by impeding MDR1 expression (Samanta et al., 2014). It is thus plausible to state that a proposed mechanism for hypoxia-induced drug resistance could operate as a positive feedback loop, where exposure to hypoxia increases MDR1 expression and administering chemotherapeutic agents further support the hypoxic environment by increasing the expression of HIF-1α and HIF-2α thereby facilitating maintenance of the drug resistance phenotype.

On the other hand, sustained activation of pro-survival pathways is seen to facilitate hypoxia induced drug resistance. In hepatocellular carcinoma, the PI3K/Akt pathway is shown to mediate HIF-1α expression consequently leading to hypoxia-induced EMT and drug resistance, which in turn facilitated increased migratory and invasive potential and impeded apoptotic induction (Jiao and Nan, 2012). Therefore, targeting HIF-1α by siRNA-silencing or treatment with a HIF-1α inhibitor restored the epithelial phenotype. Importantly, treatment with LY294002 a PI3K inhibitor significantly reduced the expression of HIF-1α, and co-administering cisplatin together with a PI3K and HIF-α inhibitor led to a significant reduction in tumor volume and increased median survival time *in-vivo* (Jiao and Nan, 2012). A different study conducted in hepatocellular carcinoma identified a role for the AMP-activated protein kinase family member 5 (ARK5) in doxorubicin resistance (Xu et al., 2016). Higher expression of ARK5 is shown to decrease doxorubicin sensitivity through EMT induction in Huh7 and Hep3B hepatocellular carcinoma cell lines. (Xu et al., 2016).

Recent studies further elucidate a role for hypoxia in the regulation anti-apoptotic proteins and in this way contributing to drug resistance. Hypoxia has been shown to increase the expression of HIF-1α and HIF-2α as well as the downstream target gene GLUT1 and further induced EMT in malignant mesothelioma, while increasing the expression of the anti-apoptotic Bcl-2 family of proteins. This consequently impeded apoptosis induction in the absence of cisplatin treatment, and also abrogated cisplatin-mediated apoptosis (Kim M.-C. et al., 2018). In colorectal cancer a HIF-1α/miR-338-5p/IL-6 feedback circuit mediates hypoxia induced-drug resistance by increasing the secretion of IL-6 and subsequently activating the IL-6/STAT/Bcl-2 pathway resulting in decreased levels of cleaved-caspase three under hypoxic conditions (Xu et al., 2019a). By impeding the HIF-1α/miR-338-5p/IL-6 feedback loop, sensitivity to oxaliplatin is restored. Interestingly, hypoxia has also been implicated in protecting cells from drug-induced senescence, where pre-exposing MDA-MB-231 cells to hypoxia mediates resistance to various classes of chemotherapeutic agents (Sullivan et al., 2008). Consequently, pre-treating MDA-MB-231 cells with an siRNA targeting HIF-1α abolishes the hypoxia-mediated increase in doxorubicin resistance and restores senescence in transfected cells to that of non-transfected cells (Sullivan et al., 2008). Studies conducted in lung cancer have identified an interesting link between the prolyl-hydroxylase domain 3 (PHD3) protein in mediating EMT, metastasis as well as drug resistance. Authors elucidated that treating lung adenocarcinoma cells with TGF-β induces EMT, increases the expression of HIF-2α and results in a significant reduction in the PHD3 protein expression (Dopeso et al., 2018).

To sum up, the mismatch between the tumor oxygen supply and consumption leads to hypoxia. Hypoxia-mediated activation of the HIF family of TFs leads to pleiotropic changes in cellular physiology thereby promoting EMT and drug resistance. The resistant phenotype is sustained through HIF-mediated regulation of the MDR transporter as well as activation of pro-survival and anti-apoptotic proteins. Based on the above findings it is evident that hypoxia mediated induction of EMT facilitates a more aggressive cancer phenotype.

Although the relationship between hypoxia and EMT has been established, no experimental data is available thus far that sheds light on the dynamics of cholesterol in this relationship. Therefore, to explore this dynamic, we propose a possible hypothesis demonstrating that it is crucial to precisely understand the role of cholesterol in cells growing under hypoxia in an attempt to target cholesterol to combat tumorigenesis.

### Proposed Model Delineating the Link Between Hypoxia, EMT and Cholesterol in Cancer

Acknowledging that cholesterol plays a crucial role in modulating cellular signaling, we propose that cellular cholesterol levels may be directly affected following hypoxia induced EMT to facilitate the acquisition of traits that promote tumor aggressiveness and drug resistance. This hypothesis is based on the evidence implicating hypoxia in the regulation of cholesterol biosynthesis through HIF-1α dependent and independent pathways (Riscal et al., 2019). Indeed, studies document hypoxia mediated translocation of SREBP2 to the nucleus thereby enhancing the expression as well as the activity of HMGCR (Pallottini et al., 2008). This is supported by studies highlighting increased levels of cholesterol in cells exposed to hypoxic conditions (Danza et al., 2013). Moreover, a study implicated the hypoxic tumor microenvironment in promoting SREBP transcriptional activity thereby enhancing the expression of downstream target genes that are crucial to mediating cancer cell survival and tumorigenesis (Lewis et al., 2015). Alternatively, hypoxia induced cholesterol accumulation is also potentiated by decreased cholesterol catabolism and reverse cholesterol transport (Cao et al., 2014).

**FIGURE 5:**
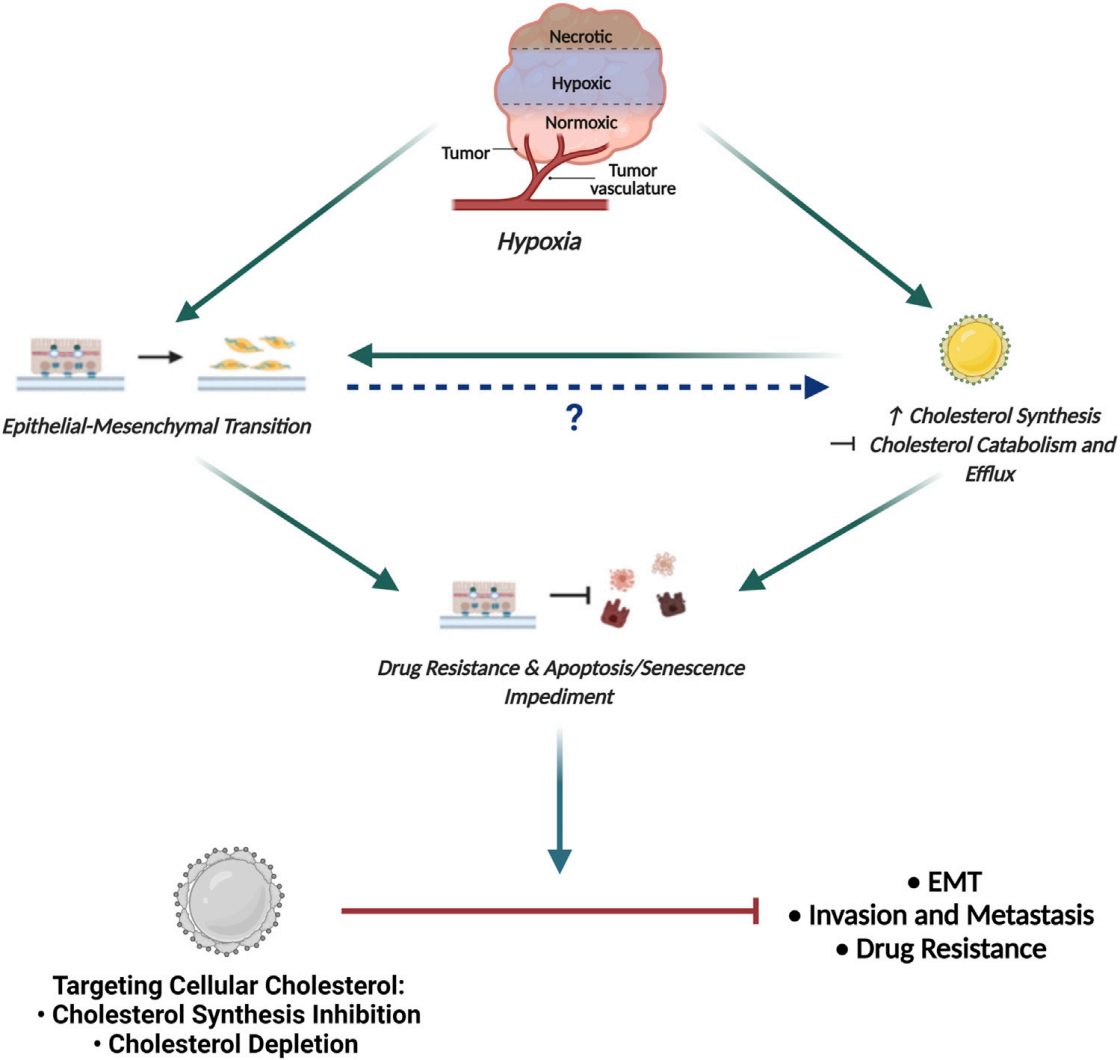
Proposed Model Delineating the link between Hypoxia, EMT and Cholesterol in Cancer. The hypoxic tumour microenvironment facilitates EMT induction and promotes increased cellular cholesterol availability by enhancing mechanisms associated with cholesterol synthesis and impeding cholesterol catabolism and efflux. While increased cholesterol content is seen to fuel EMT, how EMT affects the cellular cholesterol profile has not been delineated. Importantly, both EMT and increased cholesterol content is seen to enhance drug resistance and impede apoptotic and senescence induction. We propose that targeting cellular cholesterol, either through cholesterol synthesis inhibition or depletion, will combat EMT, decrease the invasive and metastatic potential of cells and also restore sensitivity to conventional chemotherapeutic agents.

Based on the fact that cholesterol is crucial to supporting the activity of several EMT-related protein intermediates, together with the observation of increased cholesterol following hypoxic induction, it can be hypothesized, that hypoxia-induced EMT may facilitate increased cellular cholesterol abundance by mediating both cholesterol synthesis and also abrogating cholesterol catabolism and efflux (Figure 5). Importantly, this hypothesis may be supported by studies now showing that exposing cancer cells to hypoxic conditions promotes the coalescence of lipid rafts leading to the formation of large liquid-ordered domains that facilitate compartmentalization of several proteins promoting activation of EMT-related signaling pathways (Danza et al., 2013).

By modulating lipid raft cholesterol content, EMT-associated signaling pathways are consequently affected leading to invasion and metastasis (Morandi et al., 2017). Additionally, the proposed increase in cholesterol content following EMT induction may also be implicated in altering membrane fluidity conferring cancer cells with properties necessary for cancer invasion (Morandi et al., 2017). The increase in lipid raft cholesterol following hypoxia-induced EMT is hence clearly justified, implicating cholesterol in not only directly modulating EMT associated signaling intermediates but also modifying the physical properties of the membrane to support EMT.

Furthermore, exposure to hypoxic conditions is also seen to increase lipid droplet content in cancer cells (Koizume and Miyagi, 2016). Seeing that lipid droplets serve as major storage organelles for CEs, it can be hypothesized that the increased lipid droplet content post hypoxia-induced EMT mediates an increased cellular availability of CEs. Importantly, the increase in CE lipid droplet content is also emerging as a common mechanism employed by advanced cancers to fuel cancer aggressiveness (Yue et al., 2014; Li et al., 2016). Excess of free cholesterol is toxic to cells, accordingly, storing cellular cholesterol as CEs will reduce the energetic burden on cancer cells to synthesize cholesterol *de novo*. The availability of cholesterol will further prove beneficial to the cell to facilitate membrane biogenesis and alter lipid raft dynamics thus influencing cancer cell signaling (De Gonzalo-Calvo et al., 2015). Consequently, increased levels of CEs have also been associated with increased cancer cell migration and enhanced metastatic potential (Lee et al., 2018).

Accordant with the increased cellular content, membrane lipid raft cholesterol and CEs in cells that possess an aggressive cancer phenotype, it can be hypothesized that cells post-EMT induction may display increased sensitivity to cholesterol targeting agents. This can be corroborated with findings in other studies that elucidate the beneficial outcome associated with employing cholesterol targeting agents to reduce the metastatic potential of cells by decreasing the migratory and invasive potential of cells (Murai et al., 2011; Guerra et al., 2016). This is evidenced by studies reporting that the disruption of lipid raft integrity facilitates the shedding of key cell surface receptor CD44 that cancer cells depend on for conferring an aggressive disease phenotype (Murai et al., 2011). Furthermore, several cancer stem cell and EMT related pathways are also inhibited, which facilitates an increased expression of key epithelial markers while reducing the expression of mesenchymal markers (Guerra et al., 2016; Badana et al., 2018; Sharma et al., 2019; Wu et al., 2020). It can thus be stated that cholesterol targeting agents may effectively combat EMT and metastasis and prevent the acquisition of an aggressive disease phenotype.

Seeing that cholesterol dyshomeostasis is crucial to supporting the activity of several protein intermediates in EMT-related signaling coupled with the observation of increased cellular cholesterol content in cells that have been exposed to hypoxic conditions, it can be proposed that cells post hypoxia-induced EMT should be more susceptible to cholesterol targeting agents. Consequently, targeting cellular cholesterol to combat breast cancer pathogenesis seems like a rational avenue to explore in an attempt to combat tumorigenesis (Figure 5)**.**

## Conclusion and Future Perspectives

To elucidate the crucial role of cholesterol as well as its metabolites in regulating cellular function, and how the deregulation in cholesterol metabolism mediate tumorigenesis and drug resistance is of key importance. Whereas some cancer types are dependent on increased cholesterol uptake to mediate resistance, other cancer types exploit cellular mechanisms of uptake and efflux to meet the increasing metabolic demands.

The abundance of cholesterol in addition to directly regulating protein intermediates, promotes an increase in the presence of lipid rafts which serve as key signaling hubs crucial for EMT induction. Seeing that the induction of EMT is associated with increased drug resistant potential, it can be inferred that cholesterol through its EMT-modulatory effects promotes increased drug resistance potential. Additionally, deregulated cholesterol metabolism is also seen to directly contribute to drug resistance by conferring cells with chemoresistant traits. This is achieved by cholesterol-mediated regulation of pro-survival and anti-apoptotic pathways. Additionally, based on the crucial role cholesterol plays in maintaining lipid raft integrity, evidence exists implicating lipid rafts as key domains for maintaining the activity of MDR transporters. This therefore points to the multifaceted role of cholesterol in promoting EMT and mediating cancer drug resistance.

Consequently, attempts made at targeting cellular cholesterol by employing cholesterol synthesis inhibition to restore sensitivity have been widely exploited in the laboratory setting. This has predominantly been achieved by employing the most widely used lipid lowering agents on the market, i.e. statins (Kuo et al., 2020). While employing cholesterol synthesis inhibition to reduce cellular cholesterol levels and enhance drug sensitivity has proven effective in a laboratory setting, the debilitating side effects associated with statin therapy is seen as a major limitation in a clinical setting. Common side effects include and is not limited to: muscle pains, hepato- and renal-toxicity, neurocognitive decline, myopathy as well as idiopathic polyneuropathy (Gu et al., 2019). Importantly, 30% of patient population display non-responsiveness to statin therapy (Ward et al., 2019). This could be attributed to the long-term impediment of cholesterol synthesis in cells where cholesterol plays an integral role in regulating essential cellular processes. Consequently, the discovery of new cholesterol lowering agents is pivotal to combat drug resistance. Based on the crucial role of altered cholesterol metabolism in drug resistance, screening additional compounds that either as single agents or in combination with anti-cancer therapies will restore drug sensitivity, seems like a promising avenue to pursue. Additionally, further insight is required to delineate the precise role that cholesterol plays in influencing the major components of the tumor microenvironment. This consequently highlights the incipient need to develop models that more closely mimic human immune response and the human tumor microenvironment in general. It is important to emphasize, while most of the afore-mentioned studies target cholesterol synthesis as a means to reduce cellular cholesterol levels, and consequently enhance drug sensitivity, studies conducted employing direct membrane cholesterol depletion intended for drug sensitivity restoration are lacking. Consequently, studies conducted in our laboratory currently seek to delineate the effects of direct membrane cholesterol depletion as a means to target cellular cholesterol and restore drug sensitivity and compare this approach with cholesterol synthesis inhibition strategies. By conducting these studies, a better understanding of cholesterol depletion as a strategy to restore sensitivity will be portrayed, which will further assist in addressing whether membrane cholesterol depletion rather than cholesterol synthesis inhibition could serve as a better approach to enhance drug sensitivity.

Additionally, hypoxia plays an important role in contributing to cancer drug resistance, with the major focus in this review as an inducer of EMT. Following hypoxic exposure, induction of the cellular EMT program is achieved by regulating the expression of master TFs, enhancing the production of EMT-induced inflammatory cytokines, and also regulating epigenetic mechanisms that are linked with EMT induction. It is important to emphasize that cells traverse a spectrum of intermediate states along the E-M axis and may adopt an infinite number of possible states that are dictated by the expression of various EMT markers. Interestingly, the existence in a hybrid state occurs more frequently than expected conferring upon cancer cells dynamic cellular plasticity leading to poor clinical outcome. Further experimental investigation is required to elucidate what conditions dictate whether a cell will undergo a complete EMT or exists in a hybrid state. Importantly, the establishment of molecular markers that can be utilized to distinguish the intermediate states from complete EMT is also required. This will generate a molecular signature that may assist in predicting survival and treatment response in a clinical setting. Correspondingly, studies are required to address whether a possible link exists between a specific hybrid state and drug resistance potential. Importantly, due to inherent cellular plasticity, the occurrence of both inter- and intra-tumoural heterogeneity is inevitable. This heterogeneity facilitates the existence of specific EMT hybrid states within the same niche where different areas of a tumor will be better adapted to deal with environmental pressures as opposed to areas that are not. This will serve as a platform for the selection and outgrowth of the well adapted subtypes consequently promoting metastatic dissemination and relapse. Mouse models designed to study EMT will help in addressing the identification of specific EMT states during tumor development to allow for directed targeting and eradication of resistant cell subtypes.

Furthermore, exposing cells to hypoxic conditions also contributes to drug resistance by influencing the expression and/or the activity of MDR transporters, regulating the expression of anti-apoptotic proteins, protecting cells from drug-induced senescence. Consequently, attempting to understand and delineate the integrated cell signaling biology with hypoxic conditions is crucial to better understand the beneficial outcome associated with targeting hypoxia to improve patient outcome in a clinical setting.

In conclusion, it can be stated that an intricate relationship exists between hypoxia, EMT and cholesterol that governs cancer drug resistance. While studies elucidating the link between EMT, hypoxia and resistance are well-established, studies focusing on the link between cholesterol, EMT and drug resistance are lacking. Given the importance of cholesterol dyshomeostasis in cancer initiation and progression, as well as the observation of increased cellular cholesterol in cells following EMT induction, targeting cellular cholesterol to combat breast cancer pathogenesis may be a promising direction to pursue in the search for novel anticancer therapeutics.
